# Supplementary material for: Challenging Sarcopenia: Exploring AdipoRon in Aging Skeletal Muscle as a Healthspan-Extending Shield
Source: Antioxidants (Basel). 2024 Sep 3;13(9):1073. doi: 10.3390/antiox13091073 (PMC11428238; doi:10.3390/antiox13091073)
Supplement: Supplementary file 1 [file antioxidants-13-01073-s001.zip › antioxidants-3128650-supplementary.pdf]

SUPPORTING INFORMATION

Supplementary Tables

Table S1. Circulating parameters in the three groups of mice at the end of the study.

	**Y**	**O**	**O-AR**
**Circulating parameters**			
Cholesterol (g/l)	0.86 ± 0.05	0.79 ± 0.09	0.85 ± 0.03
Triglycerides (g/l)	1.18 ± 0.14	0.76 ± 0.15	0.99 ± 0.11
NEFAs (mg/dl)	13.09 ± 1.70	10.03 ± 1.49	11.01 ± 1.00
ApN (µg/ml)	3.5 ± 0.1	3.5 ± 0.2	3.2 ± 0.2

Circulating parameters were collected from fed mice at the end of the study (at the age of 3 months for Y mice and 23 months for O/O-AR groups). NEFA, non-esterified fatty acids. Data are means ± SEM for 6Y, 6 O and 9 O-AR mice. Statistical analysis was performed using one-way ANOVA followed by Tukey's test to compare the 3 groups of mice.

Table S2. Body weight, adipose tissue weight and skeletal muscle weights in the three groups of mice at the end of the study.

	Y	O	O-AR
**Body weight (BW) (g)**	27.2 ± 0.7	35.9 ± 2.2 ***	35.7 ± 0.9 ****
**White adipose tissue weight (g)**	0.65 ± 0.04	0.75 ± 0.10	0.85 ± 0.09
**White adipose tissue weight/BW (%)**	2.39 ± 0.15	2.05 ± 0.15	2.39 ± 0.20
**Skeletal muscle weight (mg)**			
Tibialis anterior	101.8 ± 1.9	87.3 ± 4.3 **	90.6 ± 2.0 *
Gastrocnemius	325.7 ± 8.6	284.1 ± 16.9 *	295.8 ± 11.7
EDL	10.2 ± 0.4	10.8 ± 0.7	11.7 ± 1.2
Soleus	19.58 ± 0.5	17.9 ± 0.8	17.6 ± 0.8
**Skeletal muscle weight/BW (%)**			
Tibialis anterior	0.37 ± 0.01	0.25 ± 0.01 ****	0.25 ± 0.01 ****
Gastrocnemius	1.20 ± 0.01	0.81 ± 0.04 ****	0.83 ± 0.03 ****
EDL	0.04 ± 0.01	0.03 ± 0.01	0.03 ± 0.01
Soleus	0.07 ± 0.01	0.05 ± 0.01 ****	0.04 ± 0.01 ****
**Skeletal muscle weight/tibia length (mg/mm)**			
Tibialis anterior	5.84 ± 0.09	4.57 ± 0.23 ****	5.14 ± 0.09 **/ #
Gastrocnemius	18.67 ± 0.43	14.75 ± 0.74 ****	17.44 ± 0.20 ##
EDL	0.59 ± 0.02	0.61 ± 0.03	0.58 ± 0.04
Soleus	1.12 ± 0.03	0.97 ± 0.04 *	1.0 ± 0.05

Adipose tissue and muscle weights refer to pairs of fat pads (the sum of inguinal and epididymal) and skeletal muscles. Data are presented as absolute values and as a percentage of body weight. Additionally, a sarcopenia index was calculated by dividing muscle weight by tibia length. Data are means ± SEM for 6Y, 6 O and 9 O-AR mice. Statistical analysis was performed using one-way ANOVA followed by Tukey's test to compare the 3 groups of mice. *$P < 0.05$, **$P < 0.01$, ***$P < 0.001$, ****$P < 0.0001$ vs. Y mice. #$P < 0.05$, ##$P < 0.01$ vs. O mice.

Supplementary Figures

Fig. S1

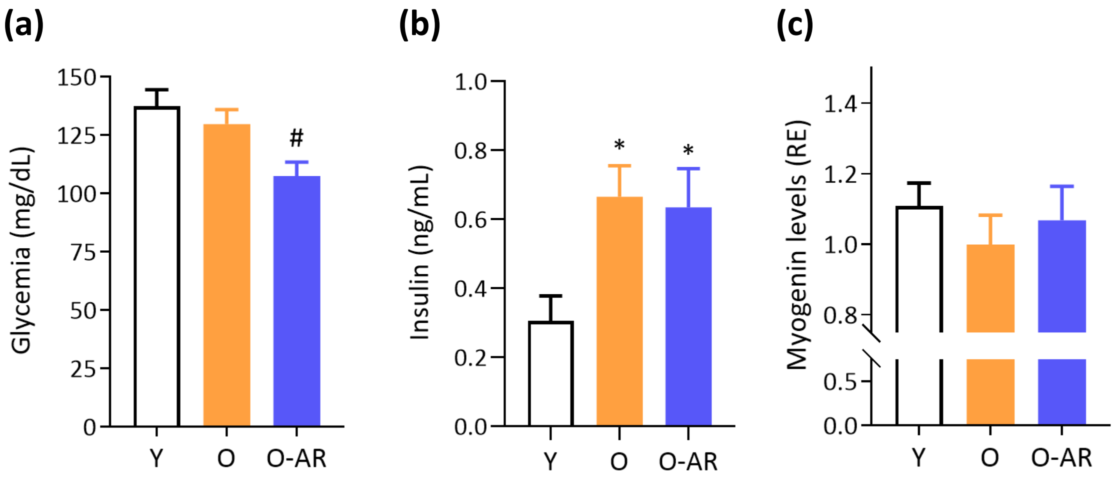

Figure S1. Glucose, Insulin and Myogenin protein levels. (a) 6h-fasting glycemia (glucometer) and (b) insulinemia (ELISA) were measured in the three groups of mice. Results are presented as mg/dl (a) and ng/ml (b). (c) Myogenin was analysed by ELISA and results are presented as relative expression compared to O values. Data are means ± SEM for 6 mice per group for all experiments. Statistical analysis was performed using a one-way ANOVA followed by Tukey's test to compare the 3 groups of mice.

Fig. S2

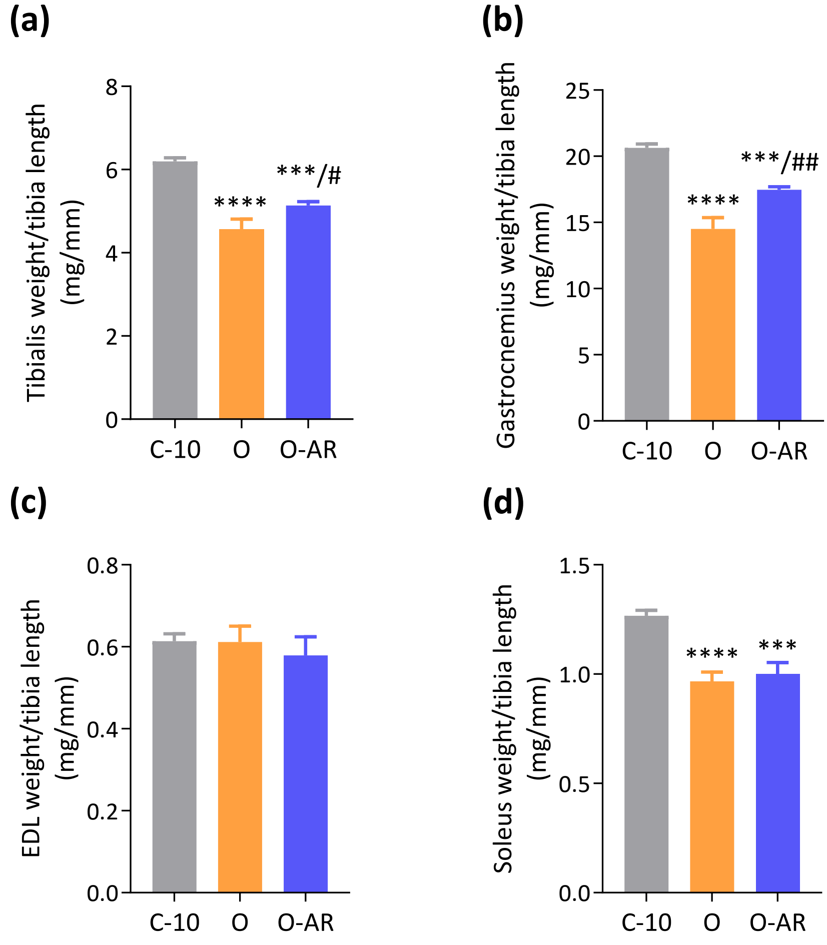

Figure S2. Sarcopenic indices for different skeletal muscles. Both groups of 23-month-old (O) mice were compared to a group of 10-month-old control mice (C-10), with similar body weight. A sarcopenia index was calculated by dividing muscle weight by tibia length. **(a)** Tibialis anterior. (**b)** Gastrocnemius. **(c)** EDL. **(d)** Soleus. Data are means ± SEM for 10 C-10, 6 O and 6-9 O-AR mice. Statistical analysis was performed using one-way ANOVA followed by Tukey's test to compare the 3 groups of mice. $^{***}P < 0.001$, $^{****}P < 0.0001$ vs. C-10 mice. $^{\#}P < 0.05$, $^{\#\#}P < 0.01$ vs. O mice.

Fig. S3

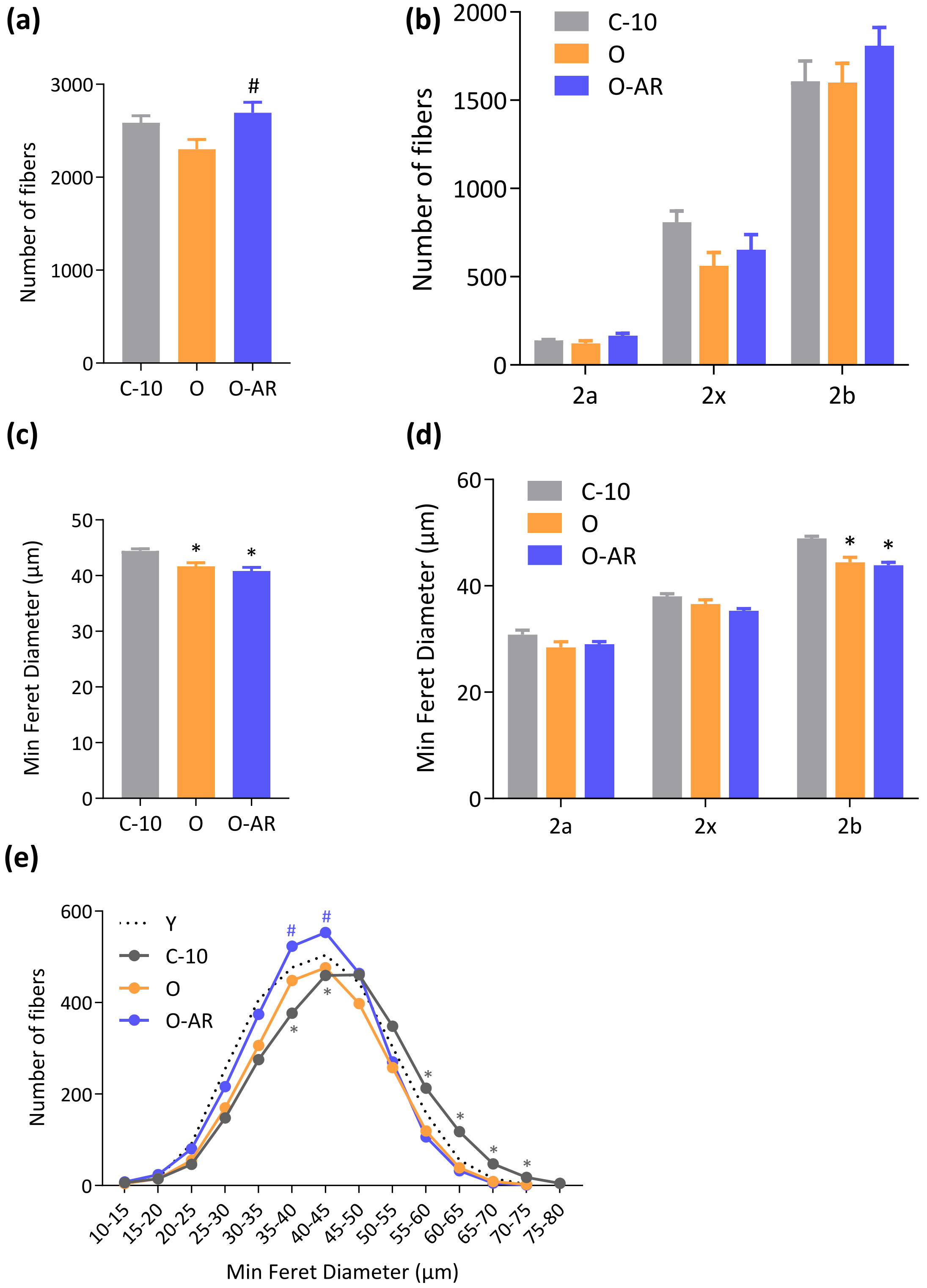

Figure S3. AdipoRon attenuates sarcopenia, but not fiber size reduction in O mice compared to 10-month-old control mice (C-10). (a) Total number of fibers and **(b)** number of each fiber type. **(c)** Global fiber size based on Minimum Feret Diameter and **(d)** fiber size based on Minimum Feret Diameter for each fiber type. **(e)** Fiber size distribution. All these data were quantified on whole cross-sections of TA. Data are means ± SEM for 4 C-10, 5 O and 6-7 O-AR. Statistical analysis was performed using one-way ANOVA followed by Tukey's test to compare the 3 groups of mice. $^{*}P < 0.05$, vs. C-10 mice. $\#P < 0.05$ vs. O mice.

Fig. S4

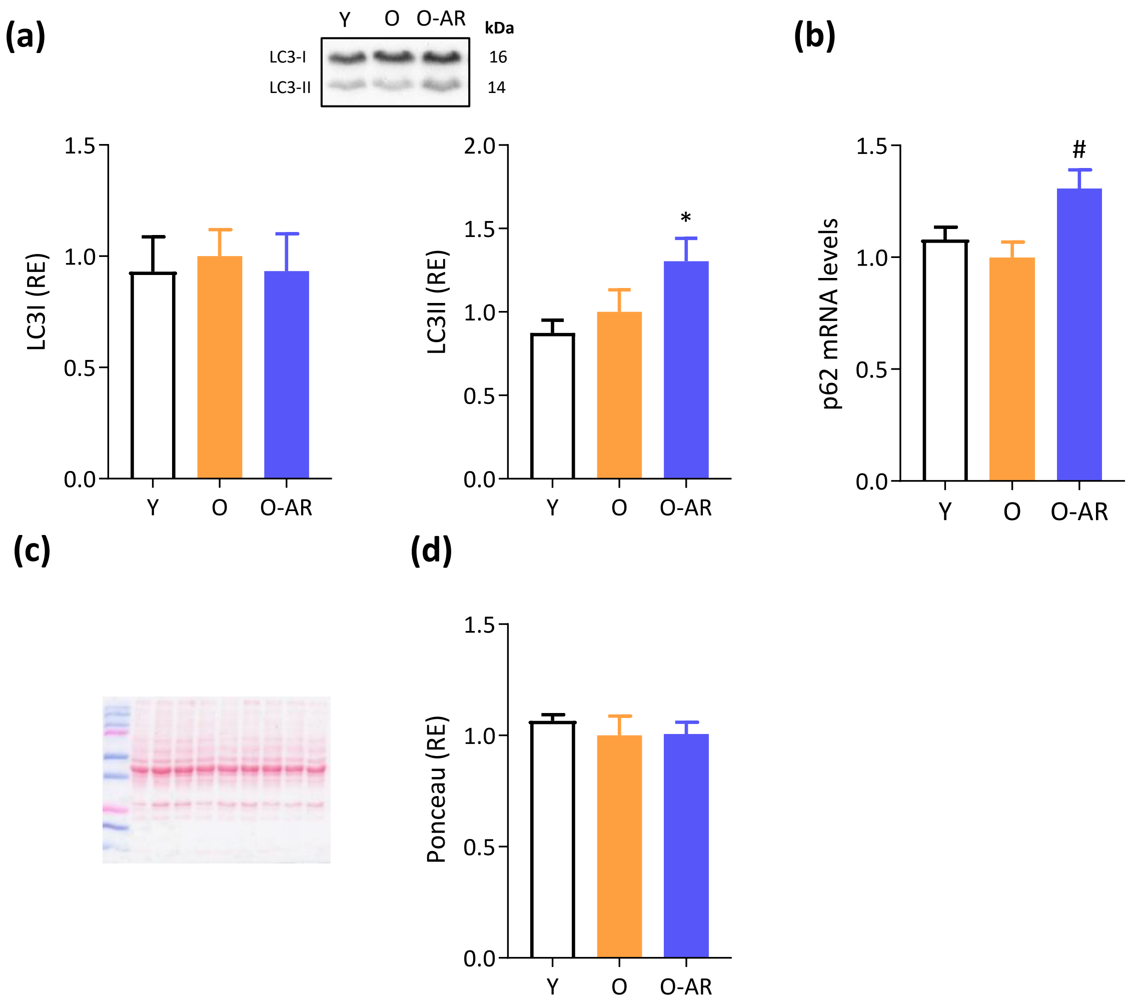

Figure S4. LC3I and LC3II protein levels, p62 mRNA levels and representative Ponceau staining. (a) LC3I and LC3II protein levels were analysed by Western blotting. **(b)** mRNA levels of *p62*, an important gene in the autophagy process, normalised to Cyclophilin. **(c)** The full ponceau-staining membrane from Western blotting with Y, O, O-AR (three animals for each condition but representative for all the animals studied). **(d)** Quantification of ponceau-staining. Results are presented as relative expression compared to O values. Data are means ± SEM for 6 Y, 6 O and 9 O-AR. Statistical analysis was performed using a one-way ANOVA followed by Tukey's test to compare the 3 groups of mice. *$P < 0.05$ vs. Y mice. #$P < 0.05$ vs. O mice.

Fig. S5

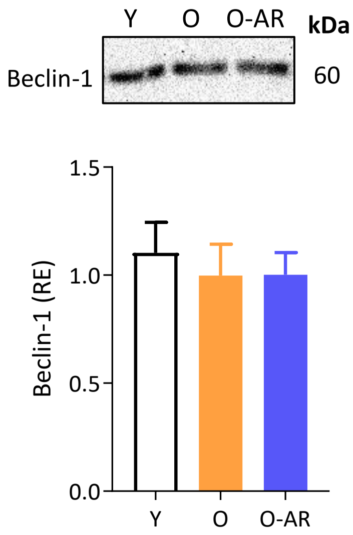

Figure S5. Beclin-1 protein levels analyzed by Western blotting. Results are presented as relative expression compared to O values. Data are means ± SEM for 6 Y, 6 O and 9 O-AR.

Fig. S6

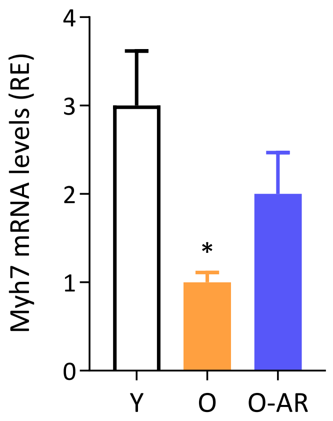

Figure S6. Myh7 mRNA levels analyzed by qPCR. Results were normalised to Cyclophilin and are presented as relative expression compared to O values. Data are means ± SEM for 6 Y, 6 O and 9 O-AR. **P* < 0.05 vs. Y mice.
